# Many roads lead to Rome: How improvisation and absorptive capacity affect entrepreneurial orientation and new venture performance relationship

**DOI:** 10.1371/journal.pone.0281456

**Published:** 2023-03-17

**Authors:** Wei Sun, Xilin Hou, Li Liang, Xiaoliang Bi

**Affiliations:** 1 School of Management, Jiangsu University of Technology, Changzhou, China; 2 School of Economics and Management, Tongji University, Shanghai, China; 3 School of Business Administration, University of Science and Technology Liaoning, Anshan, China; 4 Teaching Research Department, Urban Governance and Crisis Management Research Center, China Executive Leadership Academy Pudong, Shanghai, China; Sri Eshwar College of Engineering, INDIA

## Abstract

This paper develops and tests a model that highlights the roles of improvisation and absorptive capacity as important mediating mechanisms through which entrepreneurial orientation (EO) influences new venture performance. Furthermore, we examine the interactive effect of improvisation and absorptive capacity on new venture performance. The results show that (a) improvisation and absorptive capacity both mediate the relationship between EO and new venture performance; (b) the interaction between improvisation and absorptive capacity is positively related to new venture performance; (c) improvisation moderates the indirect relationship between EO and new venture performance via absorptive capacity; and (d) absorptive capacity moderates the mediation of improvisation in the relationship between EO and new venture performance. With these findings, this paper provides insights into how different learning modes can enhance the EO-performance relationship.

## Introduction

Under the background of economic transformation, China’s new ventures have become extensive by the country’s great support for entrepreneurial and innovative activities. New ventures, as the driving force of emerging economies, play a significant role in the sustainable development of the country. Specifically, new ventures can not only accelerate the adjustment and upgrading of industrial structure but also propel technological change, which has a critical effect on the development of region and nation [1, 2]. However, due to the liability of newness, new ventures face serious issues (e.g., insufficient legitimacy, resource scarcity), which leads to new ventures’ high risk of failure [3, 4]. More seriously, this phenomenon may even worsen under fierce competition and a complex market environment. Therefore, the survival and growth of new ventures has attracted significant attention in the field of entrepreneurship [5, 6].

Entrepreneurial orientation (EO), as a critical concept in entrepreneurship, reflects a firm’ s strategic posture to continuously engage in innovative, risk-taking, and proactive behaviors [7]. EO is a key factor in improving new venture performance, which facilitates the new ventures’ ability to identify and capture new opportunities [8, 9]. However, empirical results in prior literature on the relationship between EO and new venture performance remain inconclusive. Some studies have found that EO has a positive effect on new venture performance [10, 11], while others indicate that the relationship between EO and new venture performance is negative [12], insignificant [13], or curvilinear [14]. These different findings suggest that the linkage of EO to new venture performance is more complex than a direct effect. Therefore, there must be other factors that influence this relationship. A learning-based view has been used to explain the EO-performance relationship [15, 16]. Existing literature suggests that organizational learning factors such as learning modes can be underlying mechanisms that connect EO with firm performance [16, 17]. However, previous studies mainly focus on relatively mature firms, but fail to examine the EO-learning mode-performance configuration in the context of new ventures [16, 17]. Compared with established firms, new ventures face more severe resource constraints [18], and organizational learning has become an important way for them to acquire information and knowledge, which is conducive to the survival and growth of new ventures [19]. In addition, new ventures do not suffer from the constraints of historical knowledge and standardized procedures that established firms face [20], their learning activities may generate different effects on the EO-performance relationship for the abandonment of prior knowledge. Therefore, further research is needed to reveal the effects of organizational learning factors (e.g., learning modes) on the relationship between EO and new venture performance.

Following Hughes *et al*. [16], we suggest that improvisation and absorptive capacity may be missing links between EO and new venture performance relationship. Improvisation and absorptive capacity represent emergent and deliberate learning processes respectively [21], which exist simultaneously during the growth and maturity of new ventures [16]. Improvisation, defined as the ability to generate new combinations of resources to cope with turbulent environments, [22] describes an emergent, spontaneous, short-term, and contemporaneous learning process [23, 24]. High improvisation means rapid information collection and better decision-making for new ventures under the conditions of uncertainty and incomplete information [25], which provides support for opportunity identification, evaluation, and execution [16]. The execution of EO can compel a need for improvisation to dynamically modify the organizational actions in response to the changing environment that in turn increases firm performance [16]. Absorptive capacity, defined as the ability to value, assimilate, and apply external new knowledge [26], represents a deliberate, cumulative, and repetitive learning process [23, 27, 28]. Firms with high absorptive capacity can effectively refresh the knowledge stocks, and transform knowledge into organizational learning capability [29]. New ventures with EO can produce a lot of new knowledge in the process of entrepreneurial activities, and knowledge generated through EO can further enhance the existing absorptive capacity, which in turn improves firm performance [16]. Furthermore, Bergh and Lim [23] argued that organizational improvisation and absorptive capacity might not affect firm performance alone, but played a complementary role in creating competitive advantages. Hence, we propose improvisation and absorptive capacity are two different learning modes, and examine the way that EO affects new venture performance through the mediating and interactive effects of these two learning modes.

This study has several contributions. Firstly, this study proposes and validates an EO-learning mode-new venture performance model that explains how EO influences improvisation and absorptive capacity to achieve new venture performance, which enriches the literature on EO-performance relationship. Secondly, the organizational learning perspective has been considered as a lens to explain EO-performance relationship, which contributes to the organizational learning theory. Thirdly, we respond to the call from Bergh and Lim [23] to examine the joint effect of improvisation and absorptive capacity on firm performance, which provides empirical support for the complementary role of different learning modes in creating and maintaining sustainable competitive advantages. Finally, we answer the question of how new ventures in emerging economies can improve their firm performance through an EO, which provides an explanation of the EO-performance relationship that is different from that of developed western economies.

This paper is structured in six sections, of which the Section 1 is an introduction. The Section 2 reviews relevant literature on EO and new venture performance, and raises five hypotheses as the basis for the empirical study in this paper. Section 3 describes the research methodology. Section 4 offers analysis and results. Section 5 discusses the theoretical and managerial contributions, limitations and further research of this paper. Finally, main conclusions are drawn in the Section 6.

## Theoretical background and hypotheses

### EO and new venture performance

EO depicts an organizational decision-making process and trend regarding a firm’s key activities, strategic decisions, and managerial philosophies in the pursuit of development opportunities [7, 30]. With EO as a critical construct, scholars propose two main conceptualizations. The first conceptualization is developed from the work of Miller [31]. Miller [31] summarizes that the characteristics of startups are to engage in product-market innovation, undertake businesses with certain risks, and take proactive actions to beat competitors, based on which EO is conceptualized as the simultaneous presence of the three dimensions of innovativeness, risk-taking, and proactiveness. Lumpkin and Dess [32] propose the second conceptualization by adding two dimensions “autonomy” and “competitive aggressiveness” to the definition of EO. They reveal that the more the firm’s propensity to innovate, risk-taking, proactive, autonomous, and aggressive behavior, the stronger their EO would be. However, some scholars point out, among the five dimensions of EO, there is a certain conceptual overlap between aggressiveness and proactiveness dimension, and autonomy dimension is considered as a contextual variable in the enhancement of entrepreneurial activities [33]. Therefore, the three dimensions of innovativeness, risk-taking, and proactiveness can effectively and comprehensively reflect the connotation of EO [34].

The relationship between EO and new venture performance has gained much attention in extant literature. While prior studies on the direct relationship between these two variables have not reached consistent conclusions. A reasonable explanation for the inconsistent findings is that the relationship between EO and new venture performance is not universal, but influenced by contingencies and intervening mechanisms [5, 9]. In terms of contingencies, the moderating effects of some contingency variables have been examined, such as social capital [35], control systems [9], managerial networking [36], and legitimation [6]. As for intervening mechanisms, the mediating effects of several intervening factors have been investigated, such as environmental sustainability orientation [37], opportunity recognition [38], and entrepreneurial opportunity discovery [5].

### The mediating role of improvisation

Organizational improvisation refers to the ability of an organization to strategically change daily operations and make use of all available resources to address new environmental situations [39, 40]. Previous research suggests that EO can promote the generation of organizational improvisation to quickly respond to uncertain events [16]. Firms with a high EO will enter new product markets aggressively and incur greater risks, which means firms need to deal with more complex circumstances that emerge rapidly and unexpectedly [41]. This can further create a demand for improvisation to translate a firm’s EO (an attitudinal construct) into actions [16]. Therefore, this study proposes that EO can enhance improvisation. Specifically, the innovativeness aspect of EO can produce an organizational culture of positive reform and innovation within the firm, which can not only stimulate the enthusiasm and creativity of staff to complete tasks [42], but also improve an organization’s ability to innovate and adapt to the changing environment [43]. Thus, such an organizational climate created by a firm’s EO will contribute to organizational improvisation. In the dimension of risk-taking, risk-taking can help firms overcome the obstacles of organizational improvisation, and then improve the possibility of organizational improvisation [44]. Finally, in the dimension of proactiveness, EO’s proactiveness requires firms to grasp market trends, gain insight into market opportunities, and seize the market through advanced actions [11]. The market-leading strategic positioning puts forward higher requirements on an organization’s responsiveness in decision-making, and strategic adjustment, and prompts firms to enhance their improvisational capability.

As a form of real-time learning to cope with unexpected events under time pressure [45], improvisation plays a vital role in achieving high performance, especially for new ventures. Hmieleski and Ensley [46] found that new ventures with a tendency to improvise perform better than those less improvisational counterparts. On the one hand, improvisation will facilitate the process of information gathering and decision-making for new ventures under the conditions of uncertainty and incomplete information [25], which provides support for opportunity identification, evaluation, and execution [16]. On the other hand, improvisation usually forgoes prior knowledge and acquires information through real-time learning, which enables new ventures to make a dynamic adjustment to actions to drive superior performance [16].

Based on the above analysis, this study believes that EO can enhance new venture performance by influencing organizational improvisation. That is, organizational improvisation plays a mediating role in the relationship between EO and new venture performance. Therefore, the following hypothesis is proposed:

#### Hypothesis 1

Organizational improvisation mediates the influence of EO on new venture performance.

### The mediating role of absorptive capacity

Absorptive capacity is defined as a firm’s ability to identify, assimilate, and apply external valuable knowledge [26]. The existing literature suggests that EO as a strategic posture enables firms to develop absorptive capacity [47]. Firms with high EO will generate a lot of information and knowledge in the course of their proactive, innovative, and risky activities [16]. Continuous acquisition, assimilation, and utilization of knowledge generated by EO can further contribute to the improvement of absorptive capacity [47]. Thus, we argue that EO can enhance absorptive capacity. Specifically, firms with an innovativeness tendency are willing to increase investment in technology acquisition and product research, and pay attention to the training of employee’s knowledge and skills [34]. At the same time, they encourage employees to try innovative thoughts and methods, share knowledge and experience, and then form effective knowledge-sharing mechanisms inside the firm [34, 48]. The risk-taking tendency can increase a firm’s acceptance of new knowledge and new things, and drive employees to test old knowledge and create new knowledge by means of continuous trial and error, thereby improving the firm’s ability to assimilate, use, and create knowledge [49, 50]. Similarly, proactiveness tendency requires firms to pay close attention to the trends of policy, technology, and market, and provide timely and reliable information for discovering new opportunities [39]. In order to take effective actions ahead of competitors, firms tend to actively collect information and intelligence of customers and competitors, and absorb the tacit knowledge embedded in network relationships [51].

As an important learning mode for new ventures to overcome resource constraints [16], absorptive capacity plays a crucial role in improving new venture performance. Absorptive capacity as a knowledge-based capability enables new firms to quickly perceive and acquire information and knowledge in the external environment, and then discover and capture entrepreneurial opportunities [47]. By assimilating, transforming, and applying the acquired knowledge, absorptive capacity can further promote the development of new products and services, accelerate the creation of new knowledge, and ultimately improve the new venture’s competitiveness and performance [52, 53].

Based on the above analysis, this study argues that EO can improve new venture performance by enhancing absorptive capacity. In other words, absorptive capacity acts as a driver of EO to achieve new venture performance. Therefore, the following hypothesis is proposed:

#### Hypothesis 2

Absorptive capacity mediates the link between EO and new venture performance.

### The interactive effect of improvisation and absorptive capacity

Previous research indicates that different organizational learning modes have certain complementarity, which is reflected in the positive influence of the interaction between different learning modes on firm performance [54]. Absorptive capacity and improvisation, as two alternative learning modes, play different roles in the process of influencing firm performance [16]. Among them, absorptive capacity is the improvement of existing business activities through purposeful and planned knowledge storage [16]. Improvisation represents the ability to make immediate decisions and implement new plans through spontaneous and real-time learning when unexpected situations occur and there is not enough knowledge and experience for reference [55]. Therefore, both absorptive capacity and organizational improvisation play important roles in affecting firm performance. For new ventures that suffer from the liability of newness, absorptive capacity can be used as a learning mode to provide knowledge support for the planned production and business activities, while improvisation can be used as a learning mode to provide information basis for the adjustment of planning and decision-making made due to environmental change. Therefore, absorptive capacity and improvisation do not have contradictory influences on firm performance, but complement each other, that is, they have interactive and synergistic effects [23]. Specifically, on the one hand, new ventures with high absorptive capacity can provide experience and knowledge base for the use of improvisation through the acquisition and assimilation of external information and knowledge [23], which makes immediate decisions and bricolage made by improvisation more reasonable, and gives better play to the positive role of improvisation in opportunity identification and development. On the other hand, high improvisation enables new ventures to conduct real-time learning and rapid information collection in a dynamic environment [45], which provides new ventures with the right direction to acquire, assimilate, and apply external knowledge through absorptive capacity, thus improving the competitive advantage of new ventures. In addition, compared with established firms, new ventures are faced with more risks and challenges brought by environmental changes, for which the organizational improvisation is more likely to occur [56]. Improvisation thus plays a more important role in identifying and developing opportunities to improve new venture performance. At the same time, the absorptive capacity of new ventures can get rid of the rigid knowledge base and inflexible knowledge absorption of mature firms [16], and be more flexible and forward-looking when acquiring, digesting, and applying knowledge. Therefore, organizational improvisation and absorptive capacity of new ventures are more complementary and integrated, and the interaction between them can effectively improve performance. Based on the above analysis, we propose the following hypothesis:

#### Hypothesis 3

The interaction between organizational improvisation and absorptive capacity positively affects the new venture performance.

Above, we hypothesized that the interaction between organizational improvisation and absorptive capacity is positively related to the new venture performance, that is, absorptive capacity moderates the relationship between organizational improvisation and new venture performance, and organizational improvisation moderates the relationship between absorptive capacity and new venture performance. Combined with the hypotheses proposed before that both organizational improvisation and absorptive capacity mediate the effect of EO on new venture performance, two moderated mediation hypotheses with organizational improvisation and absorptive capacity as moderating variables and mediating variables, respectively, can be proposed. Specifically, a new venture can transfer its EO into firm performance by developing organizational improvisation, while the intervening effect of organizational improvisation can be further strengthened by the new venture’s absorptive capacity. Similarly, the presence of organizational improvisation strengthens the indirect effect of EO through absorptive capacity on new venture performance. Thus, we propose:

#### Hypothesis 4

Absorptive capacity moderates the indirect effect of EO on new venture performance through organizational improvisation, such that the mediated association will be stronger when absorptive capacity is high.

#### Hypothesis 5

Organizational improvisation moderates the indirect effect of EO on new venture performance through absorptive capacity, such that the mediated association will be stronger when organizational improvisation is high.

**Fig 1:**
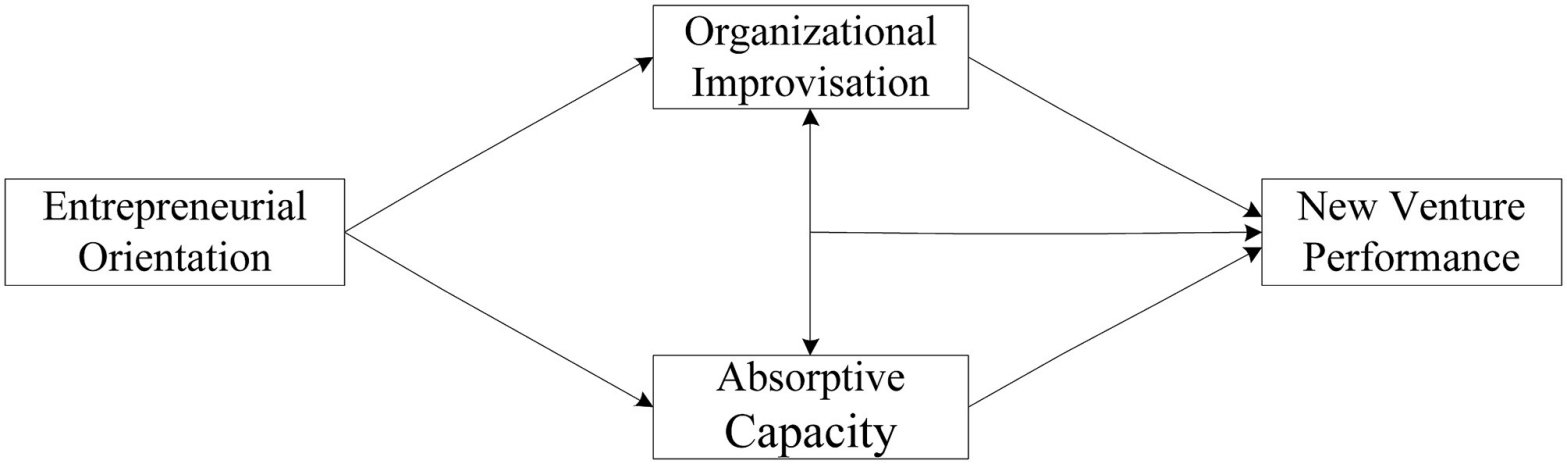
Theoretical and conceptual model.

In summary, we propose a theoretical model to unravel the mechanism of EO-new venture performance, as shown in Fig 1.

## Methodology

According to the national regulations of China, the ethical approval of the Chinese Ethics Committee is compulsory for biomedical research. Because this study was not biomedical but an organizational survey research with no minors involved, the ethical approval was not necessary.

### Sample and data collection

The survey was conducted in 2020–2021, collecting data from new ventures located in Shanghai, Jiangsu, and Zhejiang provinces in China, where the entrepreneurial activities are highly active. As one of the world’s emerging economies, China offers a rich context to explore the drivers of new venture performance. Following the process of sample selection and data collection in previous research [57, 58], we randomly selected 600 China’s new ventures within eight years old from a directory provided by the Shanghai, Jiangsu, and Zhejiang industrial associations as a sample frame.

A time-lagged two rounds of data collection were conducted to reduce the common method bias. Online and on-site interviews were conducted to collect data. The questionnaire was filled out by the new ventures’ founders because they participated in the start-up process and were familiar with the levels of EO, organizational improvisation, absorptive capacity, and venture performance. The survey was conducted by email or telephone. In the first round of the survey, the first part of the questionnaire was distributed to 600 entrepreneurs to measure EO, organizational improvisation, and absorptive capacity, of whom 425 completed the questionnaire. One year later, we conducted the second round of the survey, the second part of the questionnaire was sent to the entrepreneurs who filled out the questionnaire in the first round asking them to rate new venture performance, of whom 289 finished the questionnaire. Of the completed questionnaires, 70.59% were completed online and 29.41% were completed through on-site interviews. After excluding the invalid questionnaires, 264 valid questionnaires were finally obtained, with a response rate of 44.0%. Among the 264 new ventures, 50.0% were in high-tech industries, and 50.0% were in other industries. As for the ages of the new ventures, 17.8% have been in existence for 1–2 years, 45.1% for 3–5 years, and 37.1% for 6–8 years. In terms of firm size, 12.9% of new ventures with 10 or fewer employees, 34.5% with 11 to 50 employees, 29.9% with 51 to 100 employees, 14.4% with 101 to 500 employees, and 8.3% with more than 500 employees.

### Measures

All the scales were prepared in English and translated into Chinese according to the back-translation procedure to avoid cultural bias. The dependent variable, the two mediating variables, and the independent variables were measured with a five-point Likert scale (1 = strongly disagree; 5 = strongly agree).

### Entrepreneurial orientation

We measured EO using a nine-item scale from Covin and Slevin [7], including three components of innovativeness, risk-taking, and proactiveness. Innovativeness was measured by three items such as “Our company has launched many new products or services”. Risk-taking also was evaluated by three items such as “Our firm is more inclined to choose high-risk projects with high returns”. Proactiveness included three items such as “It is usually our company that makes the first move before our competitors respond”. The Cronbach’s alpha for EO was 0.911.

### Organizational improvisation

Organizational improvisation was measured with seven items according to Vera and Crossan [59], including creative and spontaneous two facets. Specifically, the spontaneous facet included three items such as “Employees can think and act while performing tasks”; the creative facet included four items such as “Our company often tries new ways to solve problems”. The Cronbach’s alpha for organizational improvisation was 0.911.

### Absorptive capacity

We measured absorptive capacity using the scale developed by Roberts [60]. Absorptive capacity was composed of eight items grouped into three aspects: knowledge recognition, knowledge assimilation, and knowledge application. Sample items include “Our company can identify and obtain internal and external knowledge”, “Our company has adequate procedures to assimilate new information and knowledge”, and “Our company can effectively apply knowledge to new products or services”. The Cronbach’s alpha for absorptive capacity was 0.932.

### New venture performance

We measured new venture performance using a five-item scale from Donbesuur *et al*. [5]. Respondents were requested to evaluate the extent to which their firms were successful to their competitors, regarding the number of profit growth, sales volume, sales growth, market share, and overall performance. A sample item is “Compared with competitors, our company’s profit growth was far above the competitors”. The Cronbach’s alpha for new venture performance was 0.918.

### Control variables

According to prior studies [57], we selected three control variables (e.g., firm age, firm size, and industry) that could affect new venture performance. First, firm age was measured by the number of years since a firm’s establishment. Second, firm size was measured by the logarithm of the number of hired employees. Third, the industry was defined by a dummy variable: 1 (high-tech industry) and 0 (others).

## Analysis and results

Table 1 reports the correlation coefficient, mean value, and standard deviation for each variable. Consistent with our prediction, EO is positively correlated with organizational improvisation (r = 0.193, p<0.01), absorptive capacity (r = 0.368, p<0.01), and new venture performance (r = 0.209, p<0.01). Meanwhile, organizational improvisation and absorptive capacity present positive correlations with new venture performance (r = 0.236, p<0.01; r = 0.288, p<0.01). Thus, these results are consistent with the hypotheses proposed in this study, which provides preliminary evidence for the hypothesis verification.

**Table 1:** Means, standard deviations, and correlations.

Variables	1	2	3	4	5	6	7
1.Firm age	-						
2.Firm size	0.319**	-					
3.Industry	0.110	0.153*	-				
4.Entrepreneurial orientation	0.060	0.019	0.072	(0.730)			
5.Organizational improvisation	-0.198**	-0.097	0.022	0.193**	(0.771)		
6.Absorptive capacity	0.096	0.048	0.077	0.368**	0.349**	(0.794)	
7.New venture performance	0.034	-0.107	0.136*	0.209**	0.236**	0.288**	(0.831)
Mean	4.697	3.955	0.500	3.580	3.849	3.983	3.536
SD	2.000	1.370	0.501	0.630	0.528	0.600	0.834

Note:

*p<0.05;

** p<0.01,

*** p<0.001.

The diagonal is the square root of AVE.

### Reliability and validity

First, we used Cronbach’s alpha to assess the reliability of our model. All Cronbach’s alpha values are higher than 0.9, which exceed the required benchmarks of 0.7, indicating good reliability of our measurement. Second, confirmatory factor analysis (CFA) on the main variables was conducted to test the validity of the measurement models. The results of the CFA are shown in Tables 2 and 3. As shown in Table 2, all the fitting indexes of the four-factor model are better than those of the other models (χ^2^/df = 2.024, CFI = 0.923, TLI = 0.916, RMSEA = 0.062). Therefore, the constructs of EO, organizational improvisation, absorptive capacity, and new venture performance are distinctive. As shown in Table 3, all standardized factor loadings are higher than 0.6, the AVE for each construct is greater than 0.5, and the CR of each construct is greater than 0.8, all of which are higher than the standard values. Thus, our data have good convergent validity. In addition, the square of the correlations between any two constructs is less than the AVE estimates of the two constructs, indicating that our data have good discriminant validity. Third, we conducted Harman’s single factor test on the recommendation of Podsakoff, MacKenzie [61] to calculate the influence of common method bias. The result shows that the first factor explained only 31.014% of the total variance. Therefore, our measurement does not have a serious common method bias.

**Table 2:** Model fit statistics for measurement models.

Model	χ^2^	df	χ^2^/df	CFI	TLI	RMSEA
One-factor model: EO+OI+ACAP+NVP	3305.358	377	8.768	0.408	0.363	0.172
Two-factor model: EO; OI+ACAP+NVP	2407.630	376	6.403	0.589	0.557	0.143
Two-factor model: EO+OI; ACAP+NVP	2550.209	376	6.782	0.561	0.525	0.148
Two-factor model: EO+OI+ACAP; NVP	2524.986	376	6.715	0.566	0.531	0.147
Three-factor model: EO; OI+ACAP; NVP	1615.285	374	4.319	0.749	0.728	0.112
Three-factor model: EO+OI; ACAP; NVP	1743.725	374	4.662	0.723	0.699	0.118
Three-factor model: EO; OI; ACAP+NVP	1559.614	374	4.170	0.760	0.740	0.110
Four-factor model: EO; OI; ACAP; NVP	750.756	371	2.024	0.923	0.916	0.062

Note: “+” represents two factors merged into one. EO: Entrepreneurial orientation; OI: Organizational improvisation; ACAP: Absorptive capacity; NVP: New venture performance.

**Table 3:** Results of confirmatory factor analysis.

Construct	Item	Standardized factor loading	AVE	CR
Entrepreneurial orientation	EO1	0.736	0.533	0.911
	EO2	0.713		
	EO3	0.714		
	EO4	0.720		
	EO5	0.757		
	EO6	0.724		
	EO7	0.684		
	EO8	0.760		
	EO9	0.756		
Organizational improvisation	OI1	0.759	0.595	0.911
	OI2	0.770		
	OI3	0.726		
	OI4	0.801		
	OI5	0.820		
	OI6	0.746		
	OI7	0.775		
Absorptive capacity	ACAP1	0.738	0.631	0.932
	ACAP2	0.775		
	ACAP3	0.787		
	ACAP4	0.782		
	ACAP5	0.848		
	ACAP6	0.790		
	ACAP7	0.808		
	ACAP8	0.821		
New venture performance	NVP1	0.816	0.691	0.918
	NVP2	0.862		
	NVP3	0.831		
	NVP4	0.849		
	NVP5	0.797		

### Hypotheses testing

First, the bootstrapping method was used to test the mediating effects, the results are presented in Table 4. As for the mediating effect of organizational improvisation, the results show that the indirect effect of organizational improvisation is significant (β = 0.056, 95%CI = [0.013,0.129]), and the direct effect of EO on new venture performance is still significant (β = 0.206, 95%CI = [0.050,0.363]) after adding a mediation variable. Hence, there is a partial mediating effect of organizational improvisation on the relationship between EO and new venture performance. H1 was supported. In terms of the mediating effect of absorptive capacity, the results show that the indirect effect of absorptive capacity is significant (β = 0.115, 95%CI = [0.053,0.198]), but the direct effect of EO on new venture performance is insignificant (β = 0.147, 95%CI = [-0.016,0.311]) after adding the mediation variable. Hence, absorptive capacity fully mediates the effect of EO on new venture performance. H2 was also supported. In addition, we conducted the Sobel test to further examine H1 and H2, the results confirm the mediating effects of organizational improvisation (z = 2.356, p<0.05) and absorptive capacity (z = 3.240, p<0.001).

**Table 4:** Bootstrapping and Sobel test estimates for mediation.

Path	Effects	Coefficient	SE	95% CI	z	p
EO→OI→NVP	Indirect effect	0.056	0.029	[0.013,0.129]	2.356	0.019
	Direct effect	0.206	0.079	[0.050,0.363]		
EO→ACAP→NVP	Indirect effect	0.115	0.037	[0.053,0.198]	3.240	0.001
	Direct effect	0.147	0.083	[-0.016,0.311]		

**Fig 2:**
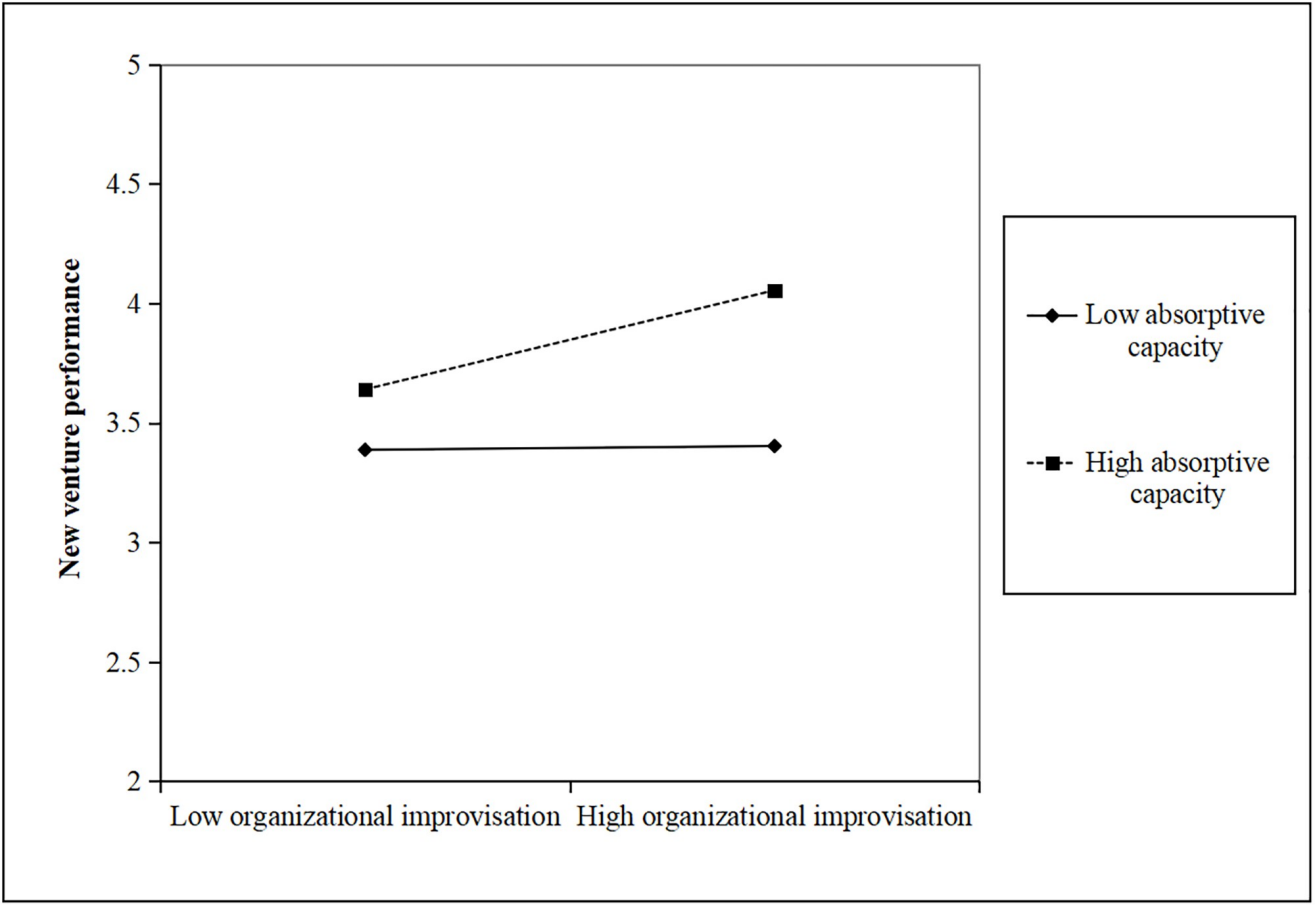
The moderating effect of absorptive capacity.

**Fig 3:**
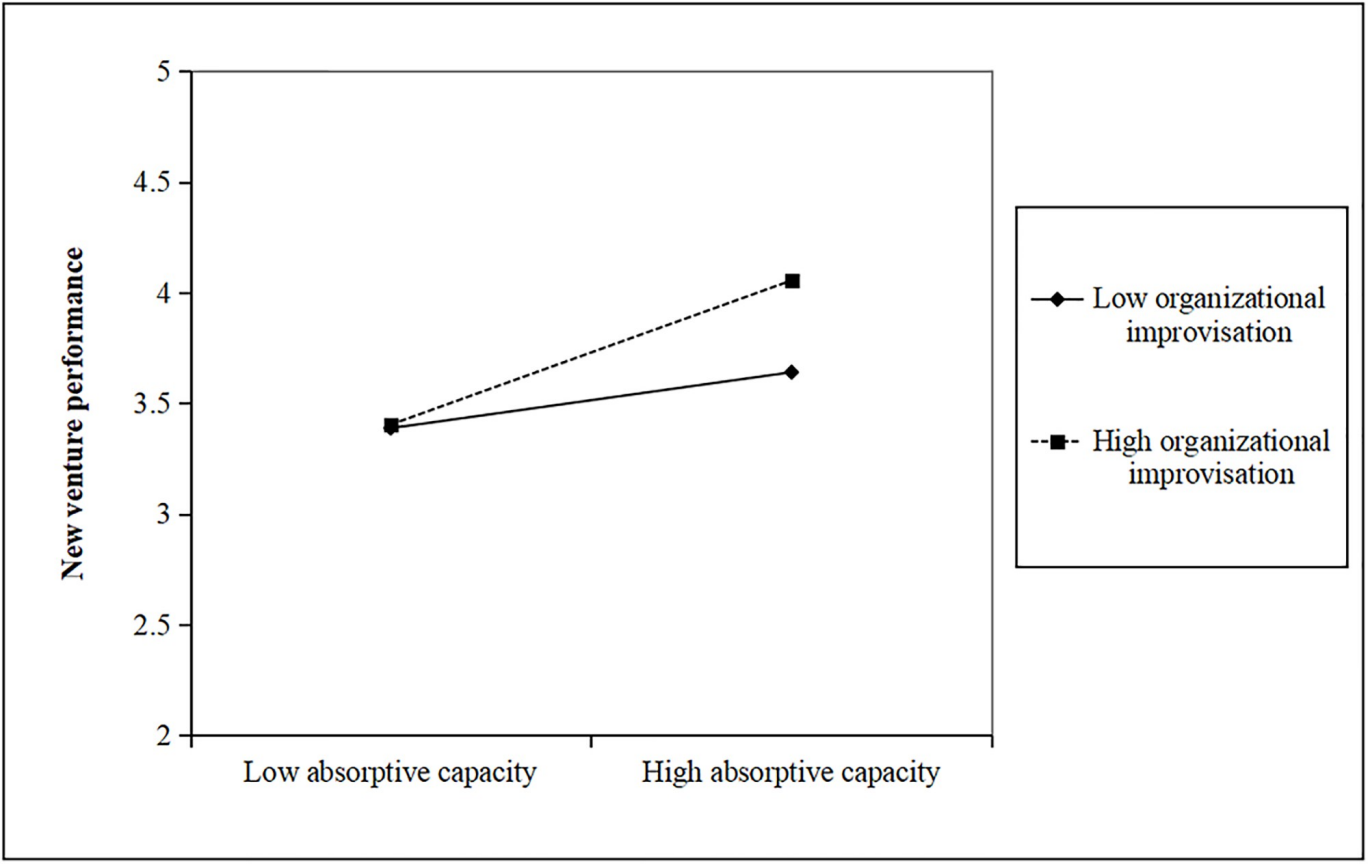
The moderating effect of organizational improvisation.

Second, we conducted a hierarchical moderated regression analysis to examine the interactive effect, with a mean-centering procedure to minimize multicollinearity, the results are reported in Table 5. As shown in Table 5, we first introduced control variables, organizational improvisation, and absorptive capacity in Model 2. Then, we added the two-way interaction term in Model 3. As shown in Model 2, both organizational improvisation (β = 0.245, p<0.05) and absorptive capacity (β = 0.311, p<0.001) were positively related to new venture performance. Results in Model 3 showed that the interaction between organizational improvisation and absorptive capacity had a significantly positive effect on new venture performance (β = 0.315, p<0.05). Thus, H3 was supported. To further research the interactive effect of organizational improvisation and absorptive capacity, we conducted a simple slope test to assess the significance of the interactive effect [62]. We first plotted in Fig 2 the effects of organizational improvisation on new venture performance for two levels of absorptive capacity. As seen from Fig 2, the relationship between organizational improvisation and new venture performance was stronger at high absorptive capacity (b = 0.393, p<0.01) than at low absorptive capacity (b = 0.015, n.s.). At the same time, we plotted in Fig 3 the effects of absorptive capacity on new venture performance for two levels of organizational improvisation. As seen from Fig 3, the relationship between absorptive capacity and new venture performance was stronger at high organizational improvisation (b = 0.543, p<0.001) than at low organizational improvisation (b = 0.211, p<0.05). Therefore, the results confirmed H3 again.

**Table 5:** Hierarchical multiple regression analysis.

Variables	New venture performance		
	M1	M2	M3
Firm age	0.027	0.032	0.034
Firm size	-0.092*	-0.089*	-0.097*
Industry	0.252*	0.215*	0.209*
IC		0.245*	0.204*
ACAP		0.311***	0.377***
IC× ACAP			0.315*
R^2^	0.039	0.137	0.151
Adjusted R^2^	0.028	0.120	0.131
ΔR^2^		0.098	0.014
F	3.514*	8.183***	7.607***
ΔF		14.634***	4.219*

Note:

*p<0.05;

** p<0.01;

*** p<0.001;

OI: Organizational improvisation; ACAP: Absorptive capacity; NVP: New venture performance.

Finally, we used bootstrapping method to test the moderated mediation effects. As shown in Table 6, the indirect effect of EO on new venture performance via organizational improvisation was significant at high (1 SD above the mean) levels of absorptive capacity (β = 0.066, 95%CI = [0.013,0.154]). However, the indirect effect was not significant at low (1 SD below the mean) levels of absorptive capacity (β = -0.003, 95%CI = [-0.082,0.062]). Thus, H4 was supported. In a similar vein, the results showed that the indirect effect of EO on new venture performance via absorptive capacity was significant at high levels of organizational improvisation (β = 0.175, 95%CI = [0.080,0.315]), while the indirect effect was not significant at low levels of organizational improvisation (β = 0.052, 95%CI = [-0.053,0.145]). Hence, H5 was supported.

**Table 6:** Results of the conditional indirect effects.

Path	Moderator level	Indirect effect	SE	95% CI
EO→OI→NVP	High ACAP	0.066	0.037	[0.013,0.154]
	Low ACAP	-0.003	0.036	[-0.082,0.062]
	Difference	0.069	0.048	[0.006,0.188]
EO→ACAP→NVP	High OI	0.175	0.061	[0.080,0.315]
	Low OI	0.052	0.049	[-0.053,0.145]
	Difference	0.123	0.077	[0.016,0.316]

## Discussion

This study advances the knowledge on the relationship between EO and new venture performance by proposing an EO-learning mode-new venture performance model. In this model, improvisation and absorptive capacity act as two key intervening factors through which EO can affect new venture performance. In addition, this study further proposes the joint effect of improvisation and absorptive capacity on new venture performance, which answers the question of what the relationship is between different learning modes in influencing the EO-performance relationship for a new venture.

Results from empirical study of Chinese new ventures suggest that both improvisation and absorptive capacity mediate the relationship between EO and new venture performance, and the interaction between improvisation and absorptive capacity is positively related to new venture performance. In addition, our findings also indicate that improvisation moderates the indirect relationship between EO and new venture performance via absorptive capacity, and absorptive capacity moderates the mediation of improvisation in the relationship between EO and new venture performance.

### Theoretical contributions

First, this study extends the EO-performance literature and contributes to organizational learning theory. The results indicate that EO’s value depends on the mediating effects of improvisation and absorptive capacity. Previous research suggests that organizational learning factors such as learning modes can be underlying mechanisms that connect EO with firm performance [16, 17], but little is known about the roles of alternative learning modes played in the relationship between EO and new venture performance. For that reason, we view improvisation and absorptive capacity as two different learning modes that co-exist in new ventures and apply the viewpoint of organizational learning to the mechanism linking EO to new venture performance. As a result, a clear picture of the EO-performance relationship is obtained that both improvisation and absorptive capacity can serve as intervening mechanisms through which EO affects new venture performance. Such findings are consistent with the results obtained by previous research that absorptive capacity plays a mediating role in the relationship between EO and firm performance [63], but inconsistent with the research indicating that improvisation is unrelated to the EO–performance relationship [16]. Therefore, this study provides a new explanation for the link between EO and new venture performance through the discovery of two parallel paths, and enriches a learning-based view of EO. Moreover, in line with organizational learning theory, we believe it is necessary to reconsider the impact of EO, with a particular focus on its role in influencing organizational learning. Adding the perspective of organizational learning helps us explain why some new ventures with resource and capacity constraints can still achieve entrepreneurial success. Last but not least, theories, predictions, and causal mechanisms of the EO-performance relationship are all derived from the studies of developed economies [64], which may lead to wrong outcomes in the theoretical expectations and predictions about the EO-learning modes-performance relationship in the research of emerging economies [16]. This study responds to the call for the research into how new ventures in emerging economies develop multiple learning modes to improve their firm performance through an EO [16], which provides an explanation of the EO-performance relationship that is different from that of developed economies.

Second, previous studies have focused on the relationship between each single learning mode and performance [16, 17, 23], but little attention was paid to the joint effect of two different learning modes on firm performance. To address this gap, this study examines the interactive effect of improvisation and absorptive capacity on new venture performance, and finds that the interaction between improvisation and absorptive capacity is positively related to new venture performance, that is, new ventures with high levels of both improvisation and absorptive capacity maximize the firm performance. This finding responds to the call for the research into how young firms manage different learning modes in the entrepreneurial activities to improve firm performance [16, 21], and answers this question with data from an emerging economy. Therefore, our study provides empirical support for the complementary role of different learning modes in creating and maintaining sustainable competitive advantages [23], and contributes to organizational learning research.

Finally, previous research calls on scholars to pay more attention to the different roles of organizational improvisation and absorptive capacity in the EO-performance relationship [16]. Responding to this call, we adopt a moderated mediation perspective to understand how improvisation and absorptive capacity influence the link between EO and new venture performance. We find that improvisation moderates the mediation of absorptive capacity in the relationship between EO and new venture performance, and absorptive capacity moderates the mediation of improvisation in the relationship between EO and new venture performance. This finding fully reveals the roles of different learning modes in the process of EO’s value creation. The adoption of moderated mediation analysis can better explain the contribution of EO to new venture performance than a mediation model.

### Managerial implications

This study also has practical implications. First, the findings suggest that improvisation and absorptive capacity significantly represent mediating roles in the relationship between EO and new venture performance, which provides new ideas for new ventures to enhance their organizational improvisation and absorptive capacity by cultivating and implementing EO strategies, and ultimately generate performance returns. As one of the world’s emerging economies, China has the most active entrepreneurial activities, and what comes with it is the ever-changing market environment. How to promote organizational learning through strategic orientation for new ventures to enhance their competitive advantage has become the key to achieving growth. Therefore, new ventures in emerging economies should pay attention to the development of organizational improvisation and absorptive capacity when making strategic plans. Specifically, new ventures, facing difficulties in legitimacy and resource acquisition, can increase their willingness to engage in innovative, risk-taking, and proactive actions by improving the level of EO, which further invites a need for absorptive capacity and improvisation as two means of knowledge acquisition, assimilation, transfer, and use [16]. The learning process produced by improvisation and absorptive capacity then enhances new venture performance.

Second, our findings regarding the complementary effect of improvisation and absorptive capacity provide some strategic guidance for new ventures. Entrepreneurs of new ventures should attach importance to the cultivation of improvisation and absorptive capacity, and strike a balance between the two learning modes. On the one hand, new ventures should pay attention to the improvement of absorptive capacity and take the development of absorptive capacity as the long-term goal of the firm. Thus, it is necessary for start-ups to incorporate the cultivation of absorptive capacity into their strategy and carry out long-term and sustained resource investment. On the other hand, new ventures should focus on the cultivation of improvisation and provide policy support and resource input for improvisational actions. This suggests that new ventures should build a learning organization, which provides a beneficial organizational environment for the generation of improvisation actions, and further increase the awareness and enthusiasm of the organization members for learning and creation. At the same time, new ventures should break some inherent patterns and conventions, provide a flexible organizational system for the generation of improvisation, and reduce the procedures of immediate decision-making and response in emergency situations.

### Limitations and future research

This study has some limitations. Firstly, our study sample is new ventures in China, which limits the generalizability of the study results to other emerging market contexts. Thus, future research might examine the relationship between EO and new venture performance across different types of emerging economies, and reveal the differences in the EO-performance relationship in different economy settings.

Secondly, there are different ways to measure new venture performance. Considering the availability of data, subjective evaluations are used to measure firm performance in this paper. Although managerial perceptions have been found to deviate minimally from objective indicators of performance, objective measures of performance are considered more valid than subjective ones [45]. Thus, future studies could adopt objective indicators to measure new venture performance.

Thirdly, the environment is an important factor that affects firm performance and the EO-performance relationship, while this study does not consider environmental contexts as an underlying mechanism. The outbreak of COVID-19, seen as a significant environmental jolt, not only brings a great impact on China’s economy, but also brings opportunity and crisis to the development of Chinese firms [65]. Thus, future research might examine the effects of the Covid-19 pandemic on strategic postures, new venture performance, and their relationships.

## Conclusion

To better explore the mechanism linking entrepreneurial orientation to new venture performance and explain the conflicting results in previous research, we apply a learning-based view to addressing the question of how alternative learning modes (e.g., improvisation and absorptive capacity) serve as key but prior-neglected mediators in the process of entrepreneurial orientation influencing new venture performance. We also reveal how improvisation and absorptive capacity interact and further influence the indirect effect of entrepreneurial orientation on new venture performance. The empirical analysis of 264 Chinese new ventures supports all the hypotheses proposed in this study. Our work and these findings contribute to a better understanding of the value creation process of new ventures’ entrepreneurial orientation, and provide a new way to explain the linkage between entrepreneurial orientation and new venture performance from learning-related processes.

## Supporting information

S1 File Questionnaire information. (DOCX)

S1 Data Research data. (XLSX)
