# Supplementary material for: Many roads lead to Rome: How improvisation and absorptive capacity affect entrepreneurial orientation and new venture performance relationship
Source: PLoS One. 2023 Mar 17;18(3):e0281456. doi: 10.1371/journal.pone.0281456 (PMC10022768; doi:10.1371/journal.pone.0281456)
Supplement: S1 File — (DOCX) [file pone.0281456.s001.docx]

**Questionnaire information**

| **Firm information** | **Option** | | | | |
| --- | --- | --- | --- | --- | --- |
| Firm size (employee number) |  |  |  |  |  |
| Firm age (year) |  |  |  |  |  |
| Industry | High-tech  industry | |  | Other industry |  |

| **Questions** | **Strongly Disagree** | **Disagree** | **Undecided** | **Agree** | **Strongly Agree** |
| --- | --- | --- | --- | --- | --- |
| **Entrepreneurial orientation** |  |  |  |  |  |
| **Innovativeness** |  |  |  |  |  |
| Our firm attaches great importance to innovation, research and development and technology leadership. | 1 | 2 | 3 | 4 | 5 |
| Our company has launched many new products or services. | 1 | 2 | 3 | 4 | 5 |
| In our firm, the improvement products or services is often a substantial innovation. | 1 | 2 | 3 | 4 | 5 |
| **Risk-taking** |  |  |  |  |  |
| Our firm is more inclined to choose high-risk projects with high returns. | 1 | 2 | 3 | 4 | 5 |
| Because of the nature of the external environment, we believe that bold, wide-ranging actions are necessary to achieve corporate objectives. | 1 | 2 | 3 | 4 | 5 |
| When faced with uncertainty in the decision-making process, the company usually takes a bold and positive attitude to maximize the potential opportunities. | 1 | 2 | 3 | 4 | 5 |
| **Proactiveness** |  |  |  |  |  |
| In a competition with its peers, it is usually our company that makes the first move before its competitors respond. | 1 | 2 | 3 | 4 | 5 |
| Our company usually takes the lead in launching new products or services, and adopting new management and operation modes, compared with its competitors. | 1 | 2 | 3 | 4 | 5 |
| In the competition with peers, our company usually adopts fierce competition to defeat its competitors. | 1 | 2 | 3 | 4 | 5 |
| **Organizational improvisation** |  |  |  |  |  |
| Employees can think and act while performing tasks. | 1 | 2 | 3 | 4 | 5 |
| Our company can immediately respond to the emergent problems in the work. | 1 | 2 | 3 | 4 | 5 |
| Our company can deal with unexpected things on the spot. | 1 | 2 | 3 | 4 | 5 |
| Our company can identify opportunities that are beneficial to its development in the new work process. | 1 | 2 | 3 | 4 | 5 |
| Our company often try new ways to solve problems. | 1 | 2 | 3 | 4 | 5 |
| Our employees are willing to take risks to come up with new ideas. | 1 | 2 | 3 | 4 | 5 |
| Our employees demonstrate originality in their work. | 1 | 2 | 3 | 4 | 5 |
| **Absorptive capacity** |  |  |  |  |  |
| Our company can identify and acquire internal and external knowledge. | 1 | 2 | 3 | 4 | 5 |
| Our company has effective procedures to identify, evaluate and introduce new information and knowledge. | 1 | 2 | 3 | 4 | 5 |
| Our company has adequate procedures to analyze the acquired information and knowledge. | 1 | 2 | 3 | 4 | 5 |
| Our company has adequate procedures to assimilate new information and knowledge. | 1 | 2 | 3 | 4 | 5 |
| Our company can successfully integrate existing knowledge with newly acquired information and knowledge. | 1 | 2 | 3 | 4 | 5 |
| Our company can effectively transform existing information into new knowledge. | 1 | 2 | 3 | 4 | 5 |
| Our company can successfully apply internal and external information and knowledge to specific applications. | 1 | 2 | 3 | 4 | 5 |
| Our company can effectively apply knowledge to new products or services. | 1 | 2 | 3 | 4 | 5 |
| **New venture performance** |  |  |  |  |  |
| Compared with competitors, our company’s profit growth was far above the competitors. | 1 | 2 | 3 | 4 | 5 |
| Compared with the competitors, our company’s sales volume was far above the competitors. | 1 | 2 | 3 | 4 | 5 |
| Compared with competitors, our company’s sales growth was far above the competitors. | 1 | 2 | 3 | 4 | 5 |
| Compared with competitors, our company’s market share was far above the competitors. | 1 | 2 | 3 | 4 | 5 |
| Compared with its competitors, our company’s overall performance was far above the competitors. | 1 | 2 | 3 | 4 | 5 |
